# Clutch Pedal Sensorization and Evaluation of the Main Parameters Related to Driver Posture

**DOI:** 10.3390/s18092797

**Published:** 2018-08-24

**Authors:** Ester Olmeda, Sergio Fuentes del Toro, María Garrosa, Jonatan Pajares Redondo, Vicente Díaz

**Affiliations:** 1 Department of Mechanical Engineering, Universidad Carlos III de Madrid, Avda. de la Universidad 30, 28911 Leganés, Spain; eolmeda@ing.uc3m.es (E.O.); mgarrosa@ing.uc3m.es (M.G.); jopajare@ing.uc3m.es (J.P.R.); vdiaz@ing.uc3m.es (V.D.); 2 Institute for Automotive Vehicle Safety (ISVA), Universidad Carlos III de Madrid, Avda. de la Universidad 30, 28911 Leganés, Spain

**Keywords:** low-cost sensors, ergonomics, health-care, force, clutch, driver position, vehicle

## Abstract

An improper decision for the design, selection and adjustment of the components needed to control a vehicle could generate negative effects and discomfort to the driver, where pedals play a very important role. The aim of the study is to provide a first approach to develop an embedded monitoring device in order to evaluate the posture of the driver, the influence of the clutch pedal and to advise about the possible risk. With that purpose in mind, a testbed was designed and two different sets of tests were carried out. The first test collected information about the volunteers who were part of the experiment, like the applied force on the clutch pedal or the body measurements. The second test was carried out to provide new insight into this matter. One of the more significant findings to emerge from this study is that the force applied on the clutch pedal provides enough information to determine correct driver posture. For this reason, a system composed of a pedal force sensor and an acquisition/processing system can fulfil the requirements to create a healthcare system focused on driver posture.

## 1. Introduction

Nowadays, there are several ways to interact with a vehicle due to the various components that are installed in it. Good design of these controls could make drivability better and more comfortable, taking into account the driver’s health. In addition, it is important to highlight that by improving these factors, pedestrian safety will improve in an indirect way.

With the advance of manufacturing processes and sensing technology, several studies use different technologies to design diagnosis or monitoring systems to avoid health problems. Mehta et al. [1] employ an accelerometer in the neck which is used to diagnose vocal hyperfunction. In [2,3] an Internet of Things (IoT) architecture is used; the first study integrates medical sensors in an IoT architecture to evaluate neonatal health at every moment. The second study proposes a distributed framework based on the IoT paradigm for monitoring human biomedical signals in activities involving physical exertion. Focusing on driver behavior, Wang et al. [4] present a method based on an electroencephalogram (EEG) to test driver fatigue, and Warwick et al. [5] present a wearable system developed in order to detect drowsiness.

The pedals of a vehicle are one of the elements that can have the most influence on driving posture. In addition, they are an essential part of the ergonomic position, which mostly depends on the driver.

Recent cases analyze the force applied on the clutch pedal in order to acquire information related to correct driver position. Table 1 shows some examples and the technology used to obtain information about the applied force by means of inertial motion units (IMU), piezoelectric sensors and strain gauges.

Nowadays, low-cost sensors are available which measure data with enough accuracy and similar performance to high-end sensors. Table 2 shows the technology used in the studies listed in Table 1 and an alternative low-cost sensor that could be used for the same purpose in each study. It is important to emphasize that the low-cost IMU accuracy was evaluated by Pajares Redondo et al. [9] with a positive result. Although this device does not measure a force value, it has the advantage of providing enough information to assess the complete pedal dynamics. Strain gauges inside the pedal sensors have high accuracy and their advantage is that they can be placed in any part of the pedal and any direction. It allows for the measurement of force distribution. In the case of piezoelectric sensors, there is no work that supports their capabilities compared to high-end sensors.

The novelty of this work is the development of a testbed that allows the acquisition of enough data from the vehicle and the driver to assess the healthiest driver position inside the vehicle in order to avoid possible future injuries or damage to the driver. This testbed allows for the acquisition of information on the characteristics of the driver (driving experience, age and height, among others) and the force applied in the clutch pedal. This testbed has been used in a real vehicle under real driving conditions.

To evaluate the viability of the system, the following hypotheses were defined:

**Hypothesis** **1.** The applied force on the clutch pedal could be used to determine if a change is necessary according to the driver’s position.

**Hypothesis** **2.** The force applied on the clutch pedal is related to the driver’s experience and age.

**Hypothesis** **3.** The horizontal distance between the driver and the clutch pedal is related to the applied force.

**Hypothesis** **4.** The actual technology to measure push forces in a vehicle could be replaced by a low-cost device.

Before explaining the methodology, it is necessary to outline some work related to ergonomics that is described in the section below.

## 2. Related Work

Some studies suggest that ergonomics and the way that a vehicle is driven can influence security, health and discomfort [10]. All of this is related to how a person is placed in the driving seat, how this person adjusts the seat settings, where the controls are placed and how much force is needed to realize the task.

Based on this, previous research on vehicles focused on the pedals. This was illustrated in the work undertaken by Jiangchuan Li et al. [7], where the authors analyzed the influence of the clutch pedal according to vehicle comfort and proposed a more appropriate separation trip. Also, a study carried out by Giacomin et al. [8] attempted to measure the comfort of the automobile clutch pedal actuator based on a certain number of experiments. Nowadays, there are other authors [6] who are focused on assessing discomfort. They are focused on the pedals, with the aim to identify and optimize different factors. This data infers that the clutch pedal plays an important role in ergonomic driver position.

MSD are defined as damages or disorders that can be caused due to a repetitive or a badly designed task, being harmful to body movements or to the musculoskeletal system (muscles, tendons, ligaments, nerves, blood vessels etc.) [11]. Some of the MSD studied by other authors are: tendonitis [12], cervicobrachial syndrome [13] and low back pain (LBP) [14], among others, although one of the most important for drivers is LBP. An example that illustrates this idea is the study developed by Bulduk et al. [15], where the authors examine the risk factors involved in WMSD (Work-related Musculoskeletal Disorders) growth in taxi drivers, finding a linked relationship between other WMSD studies. Additionally, the research by Miyamoto et al. [16] analyzes the current condition of LBP in taxi drivers. They conclude that an improvement in car seat comfort is necessary.

According to Oakman et al. [17], the factors that can increase or decrease the possibility of suffering MSD are: gender, BMI (body mass index), experience, age, exercise and physical and mental skills, among others.

For that reason, the ergonomics related to the clutch pedal and the driver’s position have been analyzed by means of a set of experiments in this article.

## 3. Material and Methods

This section introduces the experimental approach adopted to achieve the hypotheses for this work. Section 3.1 describes the experimental testbed used, taking into account that it will be replaced by low-cost devices. Section 3.2 enumerates the experiments that have been carried out for this purpose. Finally, Section 3.3 introduces the data analysis methods proposed to obtain the results.

### 3.1. Experimental Testbed Design

Three different methods have been used to obtain the required data.

1. Age, driving experience, and driver behavior are collected by questionnaires.
2. Body measurements are assessed by means of photos and weight is obtained by a scale.
3. Finally, the force applied on the clutch pedal is obtained by a pedal force sensor installed in the test vehicle which has been driven inside of a designed track.

An important consideration is that the testbed setup is the first approach in order to establish the main requirements to design a low-cost embedded system. This embedded system would be able to determine the suitability of the driver’s position to decrease injury risk and enhance healthcare for the user, based on the provided information by a low-cost force sensor.

#### 3.1.1. Questionnaires and Body Measurements

The knowledge of some of the main aspects related to the volunteers who participated in the driving test helps analyze the data and sort the results according to purpose. In terms of this idea, a first questionnaire, the body measurement, and a second questionnaire were developed.

The first questionnaire on the sizes of the subject’s limbs was designed to be filled in before starting the measurement of the applied force on the clutch pedal. Certain main classification parameters such as weight, height and driving experience were collected. More information related to this point can be found in Section 3.3.1.

The second questionnaire was used to gather information about the feelings the volunteers had throughout the experiment. The volunteers were asked to give a comfort rating and to explain the similarities between the instrumented car and their usual vehicle.

#### 3.1.2. Clutch Pedal Force Measurement

**Figure 1:**
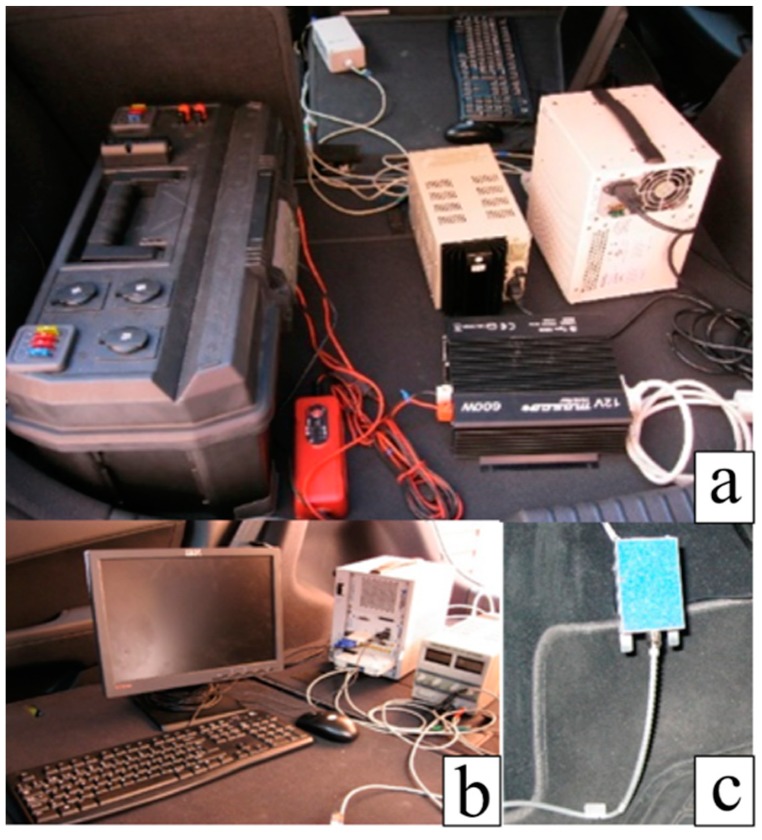
Power supply and devices used to convert the DC supply from the external battery to AC (**a**) to feed the acquisition system (**b**) and the force pedal (**c**).

To measure the force applied on the clutch pedal, the testbed introduced in Figure 1 was developed. The vehicle used was a Hyundai i30 (Madrid, Spain), where the clutch pedal was monitored using a force measuring sensor (Figure 1c) from the PK-PKH series of HKM-Messtechnik (Table 3).

This kind of sensor was chosen because it is specially designed to measure forces on pedals of vehicles. Moreover, this force measurement sensor has enough accuracy and high reliability for this work.

The data measured from the force sensor was acquired using the data acquisition system (Figure 1b) from National Instruments (NI) PXI 1031(Madrid, Spain) and the external module NI PXI 6230 (See Table 4).

Apart from this, a portable power supply (Figure 1a on the left) was designed by the mechanical engineering department of the University Carlos III in Madrid to be used in an outside test. This battery has different sockets in order to connect different devices at the same time. In the case of this study, this battery was used to plug in not only the data acquisition system, but also the force sensor, DC/AC converter and the adjustable power supply.

Table 5 shows the price of each device that completed the testbed setup and the total cost (10,265 €) is shown in the last column. Due to the high price of the complete system, the probability of it being installed on a commercial motor vehicle is very low. In view of that, this testbed setup is only a first approach in order to establish the main requirements to design a low-cost embedded system that helps to improve the driver’s position and reduces the risk of injury.

### 3.2. Tests

A first set of tests were designed to gather enough information related to the applied force on the clutch pedal, the driver and his position. All of this was collected to define the requirements of the low-cost embedded system.

After analyzing the collected data, the second set of tests were carried out. The main idea was to verify the results of the Matlab algorithm (see Section 4.3) and to prove the relationship between the horizontal distance and the force that is applied to the clutch pedal.

#### 3.2.1. First Set of Tests

**Figure 2:**
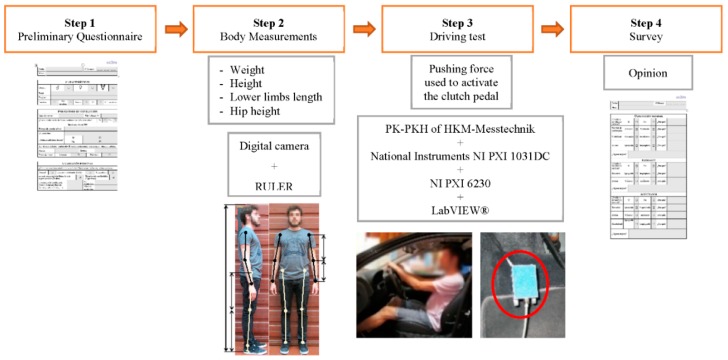
Steps each volunteer must complete in the first set of test. Information collected in every step is described together with the equipment used.

Based on the introduction of the first set of tests explained in the last section, Figure 2 shows the steps each volunteer must complete in the experiment.

- Step 1. Fill in the preliminary questionnaire, according to Section 3.1.1.
- Step 2. Measure the anthropometric dimensions of the subject (digital camera + Software RULER [18]). The anthropometric measurements of the volunteers have been carried out based on the recommendations presented by the National Institute of Workplace Safety and Hygiene (INSHT) of the Ministry of Employment and Social Security of Spain [19]. Each volunteer was marked with eighteen yellow stickers to define each limb, dimension and angle. With a camera and a certain number of photographs, twenty-three different distances of the body were measured from the frontal and profile plane. The main classification characteristics are summarized in Table 6.
- Step 3. Measure the applied force on the clutch pedal in real driving test conditions (PK-PKH of HKM-Messtechnik + National Instruments NI PXI 1031DC + NI PXI 6230 + LabVIEW^®^ [20]). The main purpose of this step was the data acquisition of the applied force on the clutch pedal (marked in red on Step 3 of Figure 2), from the footrest to the fully disengaging and engaging point. As previously mentioned, the testbed used in this research is composed of a pedal force sensor (K-PKH of HKM-Messtechnik) connected to an acquisition system (National Instruments NI PXI 1031DC + NI PXI 6230) as shown in Figure 1. The driving track was located in the facilities of the University Carlos III in Madrid. A round trip is 450 m long, with no sharp bends and an inclination of around 0%.
- Step 4. Fill in the final survey to collect the opinion regarding comfort.

#### 3.2.2. Second Set of Tests

Moving on to the second test, a new drive test to verify the results of the Matlab algorithm (see Section 4.3) was organized. This new test was designed to complete the test drive track three times with a different horizontal distance between the driver and the clutch pedal each time, attempting to prove how the horizontal distance affects the applied force.

**Figure 3:**
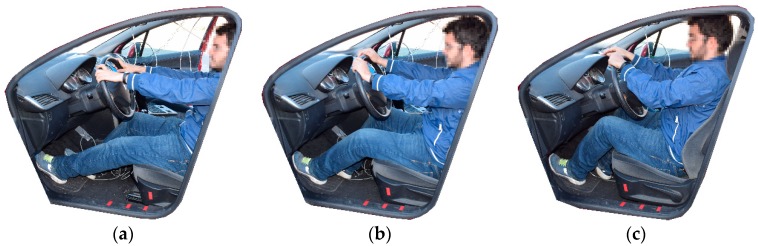
Position of the driver depending on the HC distance. (**a**) HC = 1 m, (**b**) HC = optimal distance, (**c**) HC = 0.75 m.

The three positions that have been taken into account are the following (Figure 3):

- The first position with the seat as far as possible from the clutch pedal. The distance between the clutch pedal and the hip of the driver (HC) was set in 1 m (Figure 3a).
- The second position with the optimal position assessed via the optimization tools (HC calculated using the Matlab algorithm) (Figure 3b).
- The third position with the seat as close as possible to the clutch pedal (HC = 0.75 m) (Figure 3c).

### 3.3. Data Gathering and Analysis

Turning now to the experimental results, the data that has been gathered will be presented.

#### 3.3.1. Volunteer Classification

According to the information obtained from the first questionnaire and the body measurements, the classification of the volunteers is as follows (Table 6).

**Figure 4:**
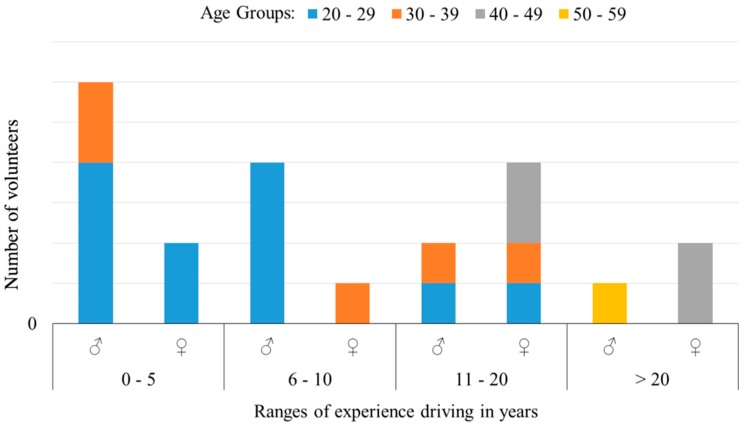
Number of people vs. driving experience, classified by age group and gender.

Also, the number of men and women who took part in the experiment and their driving experience is plotted in Figure 4.

#### 3.3.2. Applied Force on the Clutch Pedal

**Figure 5:**
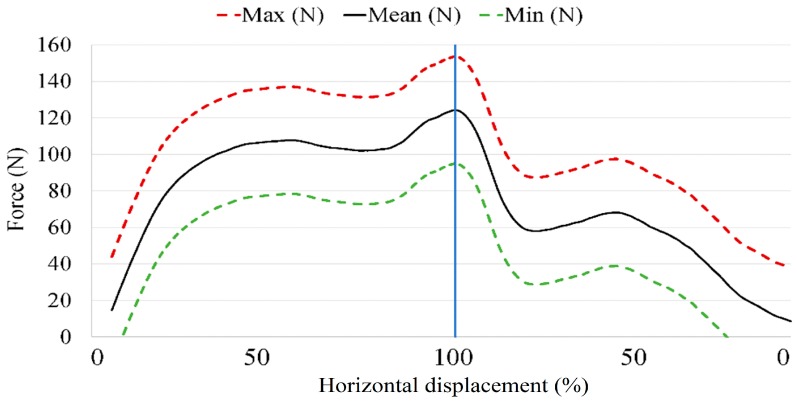
Minimum static force displacement curve of the clutch pedal (Hyundai i30). The abscissa represents the horizontal displacement of the pedal and it is divided into two sides. The left side represents clutch pedal movement from the rest position to the maximum horizontal displacement (0%→100%), and the right side from the maximum horizontal displacement to the rest position (100%→0%).

In order to be able to analyze the force curves of each volunteer that were acquired by the acquisition system during the test drives, it was first necessary to know the behavior of the clutch pedal. For that purpose, a minimum force to move the clutch was repeatedly and manually applied on the instrumented clutch pedal, which produced the following graph. (Figure 5).

Due to the different components that are part of that mechanism, the output signal of the sensor pedal shows different results for each attempt. The main dissimilarities are due to the multiple mechanical components that are part of the clutch (pedal, push rod, valve plate and spring), which have a non-linear behavior (Giacomin, Bretin 1997). For that reason, three curves have been plotted: the maximum force (red dashed line), the minimum force (green dashed line) and the average of all cases (black line). All of them represent the moving average, with the purpose of visualizing tendency. Thus, an acute hysteresis appears between the activation and deactivation force of the clutch.

**Figure 6:**
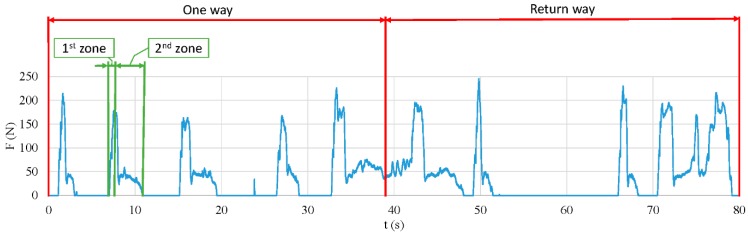
Force applied on the clutch pedal during a complete driving test. The red lines separate the curves belonging to the one way or return way direction. The green lines indicate one gear change distinguishing between the first zone (disengaging) and the second zone (engaging).

Taking that information into consideration, Figure 6 was plotted. It represents a complete curve of the forces exerted on the clutch pedal during a complete test drive. Each new peak represents a gear change. Also, Figure 6 is divided into two red areas, the one way area and the return way area.

In addition, each gear change is defined by two different zones that can be clearly distinguished because of the slope. For instance, on the left side of Figure 6, an example has been emphasized in green. The first zone goes from the baseline in the 7th second to the peak (7.8 s), and the second zone goes from the peak to the baseline (11 s).

#### 3.3.3. Rapid Entire Body Assessment Analysis

With the intention of making a first approach that would allow a simple analysis of the driving position and provide indications about the most critical factors in the driving seat, the ergonomic tool REBA was used.

This tool (REBA) is one of the most widespread methods to perform a postural evaluation, taking into account the entire body (trunk, neck, wrist, legs, etc.). In addition, it is an easy to use tool to accurately evaluate risk level.

**Figure 7:**
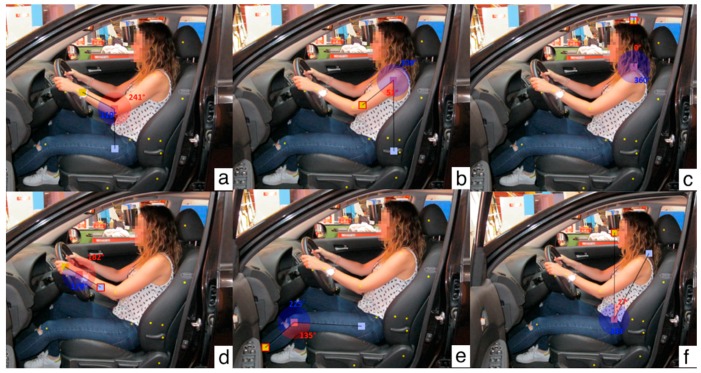
Examples of the limb angles of one volunteer. (**a**): Ankle, (**b**): Shoulder, (**c**): Neck, (**d**): Wrist, (**e**): Knee, (**f**): Trunk.

REBA [21] provides a health risk score for each posture and muscle activity, frame by frame. To achieve this, some variables are necessary. First, the angles of the body in the studied posture (Figure 7 shows an example of one of the REBA analyses carried out for this study) and second, the applied force.

The health risk score gives information about the emergence of the actions that must be carried out. According to this, the REBA score is defined by: the first level (score 1) states that no actions are required, the second level (score between 2 and 3) indicates that some actions may be necessary, the third level (score between 4 and 7) determines that in order to better define the task, corrective action and further studies are needed, the fourth level (score between 8 and 10) determines that the risk is high, so certain actions to perform the activity are necessary soon and the fifth and last level (score between 11 and 15) alerts about the need to make urgent changes.

#### 3.3.4. Matlab Model

The Matlab algorithm was designed in order to estimate the optimal horizontal distance at which the driver’s seat should be placed in order to reduce the risk of suffering any kind from damage or injury, making the driving experience more comfortable.

**Figure 8:**
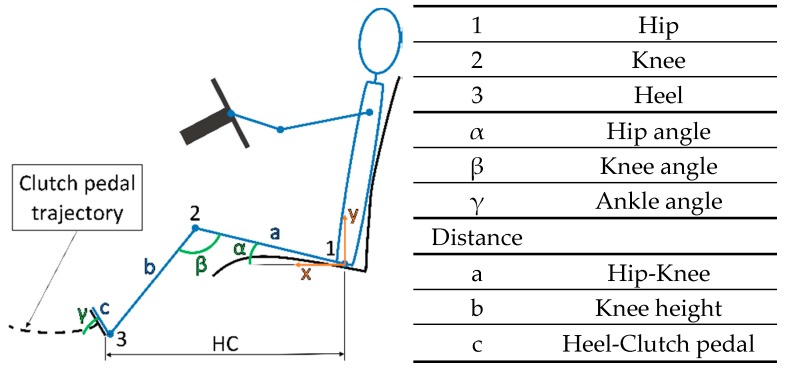
Driver seat position variables.

The different variables involved in the driver seat are defined in Figure 8.

First of all, a set of equations that define the biomechanical system of the leg were defined (1).

f(x_1_,y_1_) = f(a,α) f(x_2_,y_2_) = f(x_1_,y_1_,b,β) f(x_3_,y_3_) = f(x_2_,y_2_,c,γ) f(x_3_,y_3_) = f(γ) f(HC) = f(a,b,c,α,β,γ),(1)

These equations were included in a Matlab script in order to proceed with a trust region reflective algorithm. This algorithm provides an optimal solution for the system based on certain boundary conditions imposed on the system. Moreover, it was necessary to define the trajectory of the clutch pedal, due to the entire biomechanical system being dependent on the position in which it is located.

To obtain the trajectory of the clutch pedal, a camera recorded the position of the pedal from the origin to the footrest; therefore, the complete path was captured. After that, an analysis of several points of the pedal over the movement was developed in order to interpolate and obtain the real trajectory of the pedal.

Variables included in those limits are represented in Figure 8 and summarized in Table 7. They were selected according to the literature [22,23,24,25,26] and the ergonomic tool REBA [21].

## 4. Results

### 4.1. Applied Forces on the Clutch Pedal

**Figure 9:**
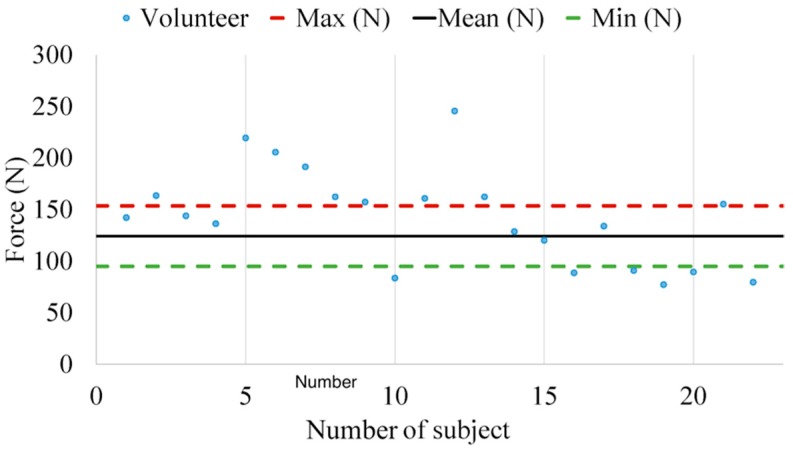
Maximum applied force distribution per volunteer.

The maximum force applied by each volunteer throughout the trial has been represented by the means of blue points in Figure 9. In addition, the maximum (red), mean (black) and minimum (green) peak values obtained in Figure 5 were plotted.

As can be seen from the data in Figure 9, not only is the distribution of these forces heterogeneous, but also several volunteers applied force outside of the margins. In particular, six out of 22 are inside the margins (red and green dashed lines), 12 out of 22 are close to the margins (±20 N) and the rest (four out of 22) applied force on the clutch pedal more than 20 N of the highest margin (red dashed line).

**Figure 10:**
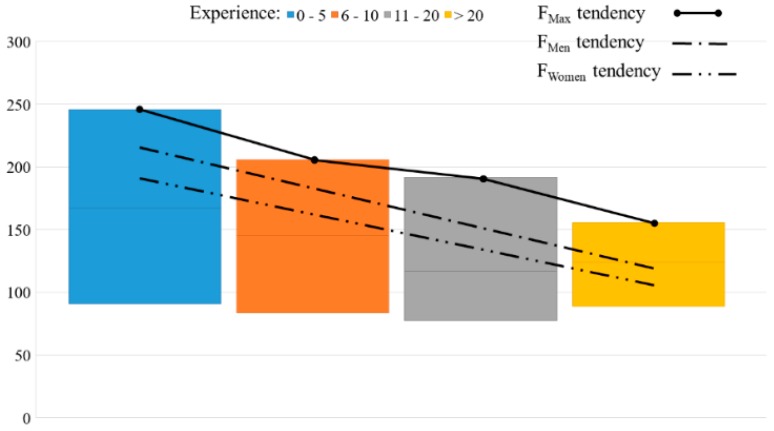
Maximum, minimum and mean force applied and force tendency of men and women according to driving experience.

The force applied by each volunteer on the clutch pedal is completely different, with the difference between the highest force and the lowest force being 166 N. In Figure 10, this difference can be seen in more detail, as well as the tendency of the force that men and women applied. Volunteers were split into four groups, according to their driving experience.

Additionally, Table 8 provides more accurate information associated with Figure 10.

### 4.2. REBA

The REBA score for each subject and position (pedal engaged and pedal disengaged) was calculated using the combination of the photos that were taken during the test (Section 3.3.3) and the angle assessment tool RULER.

**Figure 11:**
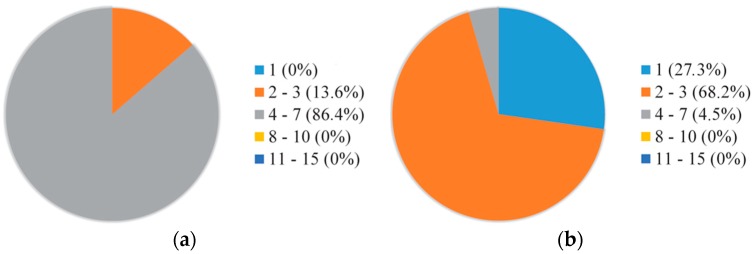
Rapid Entire Body Assessment (REBA) scores when the clutch is disengaged (**a**) and engaged (**b**).

According to the assessed results, Figure 11a represents the risk score when the driver applies the maximum force and Figure 11b represents the risk score when the clutch is engaged. As can be seen, the score risk levels for the time when the maximum force is applied are higher than for when the clutch is engaged.

### 4.3. Matlab Model

**Figure 12:**
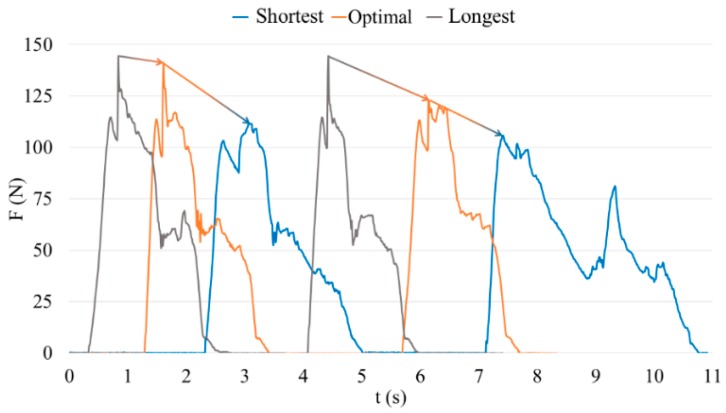
Example of the force applied by one volunteer depending on the distance of the horizontal position (HC: the shortest (HC = 0.75 m), optimal or longest one (HC = 1 m)).

Results according to the second set of tests were obtained in order to check the viability of the system. An example related to one volunteer is shown in Figure 12. This volunteer is 1.75 m tall and his knee height/hip height (KH/HH) ratio is 0.48 (KH = 0.46 m and HH = 0.95 m). The Matlab algorithm assesses that his optimal horizontal distance is 0.94 m.

In line with the explanation in Section 3.2.2, Figure 12 represents with a blue line the applied force with the shortest distance (HC = 0.75 m) between the driver and the clutch pedal, the orange line with the optimal position (HC = 0.94) and the grey line with the longest horizontal distance (HC = 1 m).

## 5. Discussions and Conclusions

Having defined what is meant by Figure 4 and Figure 10, it is necessary to determine if the drivers’ dataset accurately represents the driver population to ensure this data supports the following discussions. The range with the most volunteers is comprised of people between twenty and twenty-nine years of age. The number of women who performed the experiment was lower than the number of men (45% women and 55% men). These values are similar to the statistics provided by the National Traffic Organization of Spain database (DGT) [27]. This sample gives enough information to approach a first design, although new datasets could ensure better validity of the results.

Keeping that in mind, and with the aim of developing a low-cost monitoring system, the sample taken for the present study is considered valid for the previous approximation proposed. Also, new tests with more volunteers using the designed testbed and methodology will be necessary in order to have a bigger sample.

Concerning the REBA ergonomic tool results according to Figure 11a (maximum force is applied), most subjects in the trial (86.4%) have a REBA score between 4 and 7, which means that future studies are needed to define a strategy to adapt the mechanism. The score for the rest of the volunteers (13.6%) is lower, hence the risk is lower too, and it is not necessary to proceed with any modification.

In contrast, from the chart shown in Figure 11b, the scores related to the engaged clutch position are lower. This is mainly due to the decrease in the knee angle and force applied on the clutch pedal. The data shows that 4.5% of volunteers have a medium risk (new studies are necessary to carry out a modification), 68.2% have a low risk (actions may be needed) and finally, 27.3% have a negligible risk (action is not necessary).

Focusing on the applied force (Figure 9), what stands out is that three different groups can be distinguished. The first group is between the red and green dashed lines, the second group is close to those lines (±20 N) and the third group is comprised of people who had applied a force 20 N larger than the red dashed limit.

There was no evidence that the second group (clutch pedal ±20 N) was influenced by age or gender, because the distribution of men and women was balanced (six men and six women). On the contrary, in the third group, the driving experience was quite diverse (from two to 38 years). The results suggest that there is an association between experience and force, where people with more experience usually apply less force than people with less experience.

Following with Figure 10, it is important to highlight how the maximum force applied in every experience category is less than the maximum force in the previous one. Also, there is a decreasing tendency not only in men but also in women with a similar slope in the tendency curve. This means that there is a dependency between the force and this tendency. Furthermore, the difference between the maximum and the minimum force of the second, third and fourth range is smaller than in the first range (driving experience between zero and five years). Globally, it can be said that there is a decreasing trend, and this is probably due to the familiarity and the acquired “know how” with previous experience.

According to the second questionnaire, one of the most common complaints is that the sensitivity of the clutch pedal decreases. For that reason, one of the main premises of the device design is that it must not interfere with the normal operation of the pedal; therefore, it must be fully integrated into the mechanism.

In relation to the second set of tests, a comparison of the three curves shows clear evidence of how the force increases as the distance to the clutch pedal decreases. The evidence presents the idea that the force is directly related to the horizontal position of the driving seat. When the horizontal distance is shorter, the applied force is higher. Also, not applying a lower force implies greater comfort, but it may produce some kind of injury to the user because of the bad position. Besides the joint angles, a longer horizontal distance than necessary negatively affects posture.

As can be seen in Section 3 and as explained above, the force applied on the clutch pedal provides enough information to determine correct driver posture. For this reason, a system composed of a pedal force sensor and an acquisition/processing system can fulfil the requirements to create a healthcare system focused on driver posture. Also, the methodology that has been designed here could be used in a different kind of vehicle.

As indicated in Section 3.1.2, the testbed designed for this work can be replaced with a complete low-cost system. Not only the sensors (See Table 1 and Table 2), but also the acquisition data can be replaced with a processing system like a Raspberry Pi (about 30 €) or an Intel Edison (30 €), among others. These devices will not only acquire the input signal from the sensor, but they are also able to calculate the main parameters of driver posture in order to determine the best position to decrease the driver’s risk of suffering some kind of disease or injury. In [28], it was proved that the capabilities of the low-cost processing system are very similar to those of the high-end systems. This system can be powered by a low-voltage battery (12 €). This approach is only a first estimation and future work should be considered in order to evaluate the accuracy of the proposed low-cost system.

## Figures and Tables

**Table 1:** Comparison of the applied force on the clutch pedal between different authors.

Measured Sensors	Force (N)	Authors
Three-dimensional axis sensor	147 ≤ Force ≤ 209	Pannetier and Wang, 2014 [6]
Not defined	≈150	Jiangchuan Li et al., 2012 [7]
Two piezoelectric force sensors	124 ± 38	Giacomin and Bretin, 1997 [8]

**Table 2:** Alternative low-cost sensors proposed for each study.

Technology Used	Low-Cost Alternative	Price (€)
Three-dimensional axis sensor	BNO055	45
Two piezoelectric force sensors	7BB-20-6L0	1.60
Pedal force sensor	TE Connectivity Voltage Compression Load Cell 226.796 kg	110

**Table 3:** Technical data of the HKM-Messtechnik force sensor pedal.

**Nominal Load Range**	1500 N
**Accuracy**	0.5% full scale
**Output Signal**	0–10 V

**Table 4:** Technical data of module National Instruments (NI) PXI 6230.

**Analog Inputs:**	8 (16 bits)	**Digital Inputs:**	6
**Analog Outputs:**	4	**Digital Outputs:**	4
**Sample Rate:**	250 KS/s	**Analog Input Accuracy:**	3100 µV

**Table 5:** Testbed cost.

Device	Price (€)
PK-PKH of HKM-Messtechnik	485
NI PXI 1031	8000
NI PXI 6230	1600
Battery	80
DC/AC converter	50
Adjustable power supply	50
Total Amount	10,265

**Table 6:** Main characteristics of the volunteers.

	Age (Years)	Weight (kg)	Stature (mm)	HH */KN *	BMI	Driving Experience (Years)
Mean	30.9	71.5	1733	0.458	23.5	11.2
SD	9.8	17.5	87.9	0.034	4.1	9.5
Max/min	56/21	120/44	1854/1592	0.566/0.409	34.9/16.7	38/0.2

* HH: Hip height; KN: Knee height.

**Table 7:** Variable limits based on the completed driving test.

	Clutch Pedal Disengaged		Clutch Pedal Engaged	
	Max	Min	Max	Min
β (°)	156	135	129	82
y_2_ (m)	-	0	-	0
x_3_ (m)	1	-	1	-
y_3_ (m)	0	-	0	-
KH/HH	0.65	0.37	0.65	0.37

**Table 8:** Maximum clutch pedal force applied in every range.

	Clutch Pedal Force Applied (N)			
Experience	0–5	6–10	11–20	>20
Mean	130	177	136	141
SD	51	33	46	30
Max/min	246/77	219/142	191/80	162/120
